# Physical Activity and Sex as Predictors of Motor Development in Serbian Preschoolers

**DOI:** 10.3390/sports13100333

**Published:** 2025-10-01

**Authors:** Marko Đurović, Dušan Stupar, Emilija Petković, Ana Lilić, Vladan Pelemiš, Stefan Mijalković, Stevan Stamenković

**Affiliations:** 1 Faculty of Sport and Physical Education, University of Niš, 18000 Niš, Serbia; djura86@yahoo.com (M.Đ.); petkovicemilija@yahoo.com (E.P.); analilic93@gmail.com (A.L.); stefimijalkovic@gmail.com (S.M.); stevajudo@yahoo.com (S.S.); 2 Faculty of Sport and Psychology, TIMS, Educons University, 21000 Novi Sad, Serbia; 3 Faculty of Education, University of Belgrade, 11000 Belgrade, Serbia; vladan.pelemis@uf.bg.ac.rs

**Keywords:** Body Coordination Test for Children, abilities, physical fitness, motor competence

## Abstract

**Background/Objectives**: Motor coordination is the essential ability that influences children’s overall physical development and their ability to engage in various activities. The development of motor skills and coordination continues for several years, as it is a gradual process that extends beyond the early stages of walking. The study aimed to describe the differences in genders and physical activity levels using the Body Coordination Test for Children (KTK) test battery to assess motor coordination in kindergarten children. **Methods**: The sample consisted of 814 participants, including both sexes (aged 5.57 ± 0.49 years). Participants were divided into two groups according to their involvement in physical activity (OPA) or physical inactivity (NO OPA). OPA had to have a minimum of 2 days per week of additional organized training/lessons with at least 60 min. **Results**: The results show significant gender differences in walking backwards; girls outperformed boys with a statistically significant mean difference of −3.11 (*p* = 0.01; 95% CI: −4.57 to −1.64). Similarly, for total motor quotient (MQ), girls scored higher than boys, with a significant mean difference of −4.92 (*p* = 0.01; 95% CI: −7.85 to −1.99). The results revealed that the OPA group consistently outperformed the NO OPA group across all subtests, with significant differences in Total MQ (*p* = 0.01). **Conclusions**: Our study results indicated that physically active children demonstrated better motor coordination in comparison to their inactive peers. This suggests that regular physical activity positively influences motor coordination development in children.

## 1. Introduction

Even after children master the complex skill of walking independently, their movements remain less coordinated compared to adults. In reality, the development of motor skills and coordination continues for several years, as it is a gradual process that extends beyond the early stages of walking [1]. This ongoing development is essential for achieving full motor proficiency. Motor coordination is a fundamental skill that influences children’s overall physical development and their ability to engage in various activities [1,2]. Clark and Metcalf [3] emphasize that the acquisition of basic motor skills is crucial, as deficiencies in locomotor and manipulative abilities can significantly limit children’s opportunities to participate in physical activities as they grow. Research suggests that participation in diverse physical activities can significantly enhance motor coordination, while inactivity may hinder its development [4]. Furthermore, motor coordination is a key factor in performing complex movements that involve multiple body parts and spatial awareness, which are essential for both daily life and sports performance [5].

Several studies highlight the importance of motor coordination in early childhood, particularly in relation to physical activity and long-term health outcomes. Research on physical activity shows that gross motor competence is linked to various health outcomes. Children who have low gross motor competence are usually less physically active and possess lower cardiorespiratory fitness levels and lower motor abilities [6]. Lopes et al. [7] suggest that developing motor coordination should be a strategic priority for obesity prevention and the promotion of physical activity in children. Moreover, Lubans et al. [8] establish a positive correlation between motor competence and overall health, reinforcing the significance of structured motor skill development programs.

So far, various motor test batteries have been created to assess and track motor competence at different stages across the lifetime. These tests help researchers and practitioners understand motor development and identify potential delays or issues early in growth. To assess motor coordination, researchers frequently use the Body Coordination Test for Children (Körperkoordinationstest für Kinder—KTK) test battery [9]. The KTK is widely regarded as a reliable instrument for evaluating fundamental motor skills, dynamic balance, and gross motor coordination in children aged 5–14 years [10,11]. Its applicability extends beyond sports and education into health sciences, biomechanics, and medicine, demonstrating its versatility [9]. Also, it indicates the capacity to perform a broad array of physical movements that involve total body coordination [12]. However, despite its widespread use, limited studies have validated the reliability of the KTK in specific populations [9]. The KTK has been widely used in research to study motor development, physical fitness, and the effects of interventions on children’s coordination. For example, studies have explored its reliability and validity across different populations, including children with typical and atypical development [9]. Additionally, research investigating the effects of targeted interventions on motor outcomes assessed by the KTK remains scarce [13,14].

While motor coordination develops naturally through maturation [1], optimal coordination levels are best achieved through structured training. Fransen et al. [15] highlight that participating in diverse sports activities significantly enhances gross motor coordination. Jaakkola et al. [16] further emphasize that sport-specific training can improve motor coordination, though its effectiveness depends on the type of sport in which children engage. Popović et al. [17] those involved in multiple sports were observed to have superior motor coordination skills to those who specialized in a single sport, such as football. The findings highlight the importance of multisport engagement in fostering well-rounded motor skills, which can have lasting benefits for children’s physical health and athletic potential [4]. Overall, when participants complete an MC test battery, girls typically achieve higher scores in the balance test, while no significant differences are usually seen between boys and girls in locomotion tests [18,19]. Nevertheless, these results are mostly seen in the younger age groups. However, there is still a lack of research comparing the differences in gender on motor coordination, particularly regarding sports, with the KTK test. Given the different sports academies, schools, and clubs’ popularity among children and their early engagement trends, it is essential to explore their role in motor coordination development. Efficient sports activity requires precise synchronization of limb and body movements, further underlining the importance of motor coordination.

A review of the existing literature reveals a lack of research into differences in motor coordination by gender and physical activity level in preschoolers. Researching motor coordination across different genders and physical activity levels is essential for understanding how biological and environmental factors influence physical performance, development, and overall health. Therefore, the present study aimed to describe the differences in genders and physical activity levels using the KTK battery test to assess motor coordination in kindergarten children. The exact dimensions of physical activity, such as intensity and frequency, were planned and programmed in training sessions in sports clubs or sports schools with moderate or vigorous activity. Based on the outlined aim and the existing gap in the literature, it is assumed that preschool children engaged in organized sports activities will demonstrate higher levels of motor coordination, as measured by the KTK battery, compared to their peers who are not involved in such programs. Furthermore, it is expected that differences in motor coordination will also be observed between boys and girls, highlighting the role of both physical activity and gender in shaping motor development at an early age. The results of this study would contribute to better showing the role of gender differences in motor coordination and different physical activity levels, and in this way, teachers, parents, etc., receive appropriate information to maximize children’s motor potential and long-term well-being.

## 2. Materials and Methods

### 2.1. Participants

The sample of 814 participants, including both sexes (boys = 409; girls = 405) aged 5.57 ± 0.49 years and with a BMI of 17.16 ± 21.2 in this study, was studied with a cross-sectional design. Participants were split into two groups according to their participation in organized extracurricular physical activity or no organized extracurricular physical activity. Inactive children (NO OPA) included in the study were not involved in any additional organized physical activity beyond mandatory physical education in kindergarten and were from the territory of southern Serbia (boys = 226; girls = 244). The second group, the physical activity group (OPA), included children who had been engaged for at least six months in some sports school or sports club (boys = 175; girls = 150). Additional inclusion criteria were that OPA children had to have a minimum of 2 days per week of additional training with at least 60 min per training. The participants were included in regular kindergarten education in the territory of Serbia, where they had the opportunity to participate voluntarily in the measurement. Also, only children enrolled in city kindergartens were included in the study to maintain a homogeneous sample in terms of setting and access to facilities. The excluded children had a motoric disability or were children who could not finish all subtests included in the KTK test battery.

All parents and their children were first informed about the study. Written informed consent was received from the children’s parents or guardian(s). The purpose and aim of the study were explained to them, along with any possible consequences. Ethical approval for the study was obtained from the institutional ethics committee of the University of Niš (ref no.: 04-2018/3). Before the testing protocol, parents or guardians must sign a consent form for their children’s participation.

### 2.2. Procedures

Prior to the testing process, parents are required to complete a detailed survey regarding their children’s physical activity habits. This survey gathers information about the type and frequency of sports and activities in which the children participate, encompassing both team sports like football, basketball, and athletics, as well as individual pursuits such as artistic gymnastics, judo, and swimming. For a child to be considered physically active, they need to engage in such activities at least two days per week, dedicating approximately 60 min per session. Standardized protocols were used to assess height—it was measured using an anthropometer (Seca 220; Seca Corporation, Hamburg, Germany) to the nearest 0.1 cm—and body mass was measured using a portable Omron BF-511 [20] scale to the nearest 0.1 kg. Body mass index (BMI) was calculated using the formula BMI = $\frac{Weight\;in\;kg}{Height\;in\;m^{2}}$. The testing was managed by two PhD students and two professors [21]. The tests took place in the kindergarten gym during the morning and were completed in a single day. The testing schedule began with taking anthropometric measurements—specifically, body height and weight—for all children. Afterward, the children were split into four groups, each starting at a different KTK subtest: walking backward on beams, lateral jumps, hopping on one leg over obstacles, and moving platforms. Once a group finished its assigned test, it rotated clockwise according to a prearranged schedule. The testing finished once all groups had completed all four subtests. The stations were set up in the corners of the gym to allow for a logical flow of group movement.

### 2.3. Measures

The KTK consists of 4 subtests: (1) walking backwards along a balance beam, (2) jumping sideways, (3) moving sideways on boxes, and (4) hopping on one foot [22]. Motor Quotient (MQ) is a standardized indicator that allows a child’s motor performance to be evaluated not only through the number of points achieved or repetitions performed, but in relation to age- and gender-specific norms [22]. A motor quotient (MQ), adjusted for age and gender, was obtained from these four subtests using reference data from 1128 healthy German children [22]. The “motor quotient” (MQ), a global indicator of MC adjusted for age and gender, was calculated using the four items and used as an indicator of MC. The MQ allows an assessment of the gross motor development in the following categories: ‘severe motor disorder’ (MQ 56–70, percentile 0–2), ‘moderate motor disorder’ (MQ 71–85, percentile 3–16), ‘normal’ (MQ 86–115, percentile 17–84), ‘good’ (MQ 116–130, percentile 85–98), and ‘high’ (MQ 131–145, percentile 99–100).

#### 2.3.1. KTK Subtest Descriptions, Equipment Needs, and Scoring

##### Walking Backwards

This subtest assessed balance, rhythm, and strength. These three motor skills reflect a child’s ability to sustain postural control while moving backward on balance beams of three different widths. The first beam measures 6.0 cm, the second 4.5 cm, and the third 3.0 cm in width. Each beam has a total length of seven feet.

Scoring Guidelines. Before starting the WB subtest, children remove both shoes and socks. They are instructed to walk backward across the three balance beams (6.0 cm, 4.5 cm, and 3.0 cm). On each trial, children can take up to eight steps, giving a maximum of 24 steps per beam and 72 steps across all three beams. Each child is allowed three trials per beam in an attempt to reach the maximum of eight steps. The test begins with the child walking forward along the beam until stepping onto the wooden platform. The backward walk then starts from this point. The initial step backward from the platform onto the beam is referred to as the plantar step and is not included in the scoring. Every successful backward step counts as one point, with up to eight points possible in each trial. If the child falls or touches the ground with any part of the body, the trial is stopped, and the next attempt begins again from the wooden platform. After completing three trials, the child proceeds to the next beam width and repeats the same procedure.

##### Jumping Sideways

This subtest evaluates speed, rhythm, and agility. Together, these physical skills determine how well a child can move laterally and maintain postural control while continuously jumping side to side for 15 s. The lateral jump task is performed using a wooden obstacle measuring 5 ft 10 in in length and 24 in in width, with a vertical wooden barrier sized 25 cm × 25 cm × 5.7 cm (L × W × H).

Scoring Guidelines. Before beginning, the child stands with both feet flat on the ground to the right of the wooden divider. The task is to jump laterally over the divider as many times as possible within 15 s. For a jump to be counted, the child must avoid making contact with the divider and land with both feet touching the ground at the same time. Each valid jump is worth one point. Since the test is time-based, there is no set maximum number of jumps; instead, children are encouraged to achieve their highest possible score within the 15 s period. Each child completes two trials, and the total score is calculated by summing the results from both attempts.

##### Moving Sideways

This subtest evaluates children’s ability to move laterally across a wooden platform using a repeated crossover motion. The SS subtest measures speed, rhythm, strength, and balance. These physical components together indicate how effectively a child can step sideways while maintaining postural control during a 20 s period of continuous lateral stepping. The test requires two wooden platforms, each measuring 25 cm × 25 cm × 5.7 cm (L × W × H).

Scoring Guidelines. At the start, the child stands on one of the wooden platforms. They then lift the second platform and place it to their left or right, crossing one leg over the other in a lateral crossover step. The child moves onto the newly placed platform and repeats this crossover stepping pattern for 20 s, with the goal of completing as many successful movements as possible. Each child is given two trials, with a mandatory 10 s rest between attempts to recover before continuing. Scoring is based on two components: one point is awarded for each crossover movement performed (1 pt = crossover), and another point for successfully stepping onto the platform after the crossover (1 pt = step). As this is a timed subtest, there is no fixed maximum number of steps, so children are encouraged to perform as many as they can. After both trials are completed, the scores from each attempt are combined and recorded.

##### Hopping for Height

This subtest evaluates each leg separately by having the child hop over a foam barrier that increases in height by 5 cm after every successful jump. It measures strength, rhythm, and balance, which together indicate how explosive one leg is in achieving a controlled landing over progressively higher obstacles. After each successful jump, an additional 5 cm foam pad is added until the child fails to clear the obstacle within three attempts. A trifold mat is required for safety, and the evaluator must ensure a clear pathway of at least six feet. The test requires twelve foam pads, each measuring 60 cm × 20 cm × 5 cm (L × W × H), stacked progressively to reach a maximum height of 60 cm. The objective is for the child to clear the full height with each leg.

Scoring Guidelines. Before starting, children remove their socks and shoes. Standing just off the trifold mat, the evaluator designates which leg is being tested. The child balances on that leg, then attempts to hop over the foam obstacle, landing on the same leg and performing two additional hops after landing for the attempt to count. If the non-tested leg or any other body part touches the ground, or if the two follow-up hops cannot be completed, the attempt is invalid. Each leg (right and left) is given three attempts per height. Scoring depends on when the successful attempt occurs: three points for success on the first try, two points for the second, and one point for the third. If no successful hop occurs within three attempts, the child scores zero for that height, indicating they have reached their JH ceiling, and no further increases are attempted for that leg. The evaluator then repeats the same process for the opposite leg, raising the obstacle height in 5 cm increments up to 60 cm (5 cm, 10 cm, 15 cm, etc.). The test ends when both legs are unable to clear the obstacle within three tries. The maximum possible score is 72 points, achieved if the child clears all twelve foam heights on the first attempt with each leg, earning three points per height.

### 2.4. Statistical Analysis

The data are presented as means ± standard deviations. Prior to applying parametric tests, the normality of the data was assessed using the Kolmogorov–Smirnov test. Differences between groups and between genders were analyzed using an independent *t*-test. A significance level of *p* < 0.05 was applied. All statistical analyses were conducted using SPSS software. (SPSS 26.0, IBM Inc., Chicago, IL, USA). To account for differences in age and gender, group differences between preschoolers were examined using a one-way ANCOVA. Additionally, Cohen’s d effect sizes with 95% confidence intervals (95% CI) were calculated to evaluate the practical relevance of these differences, using the following thresholds: 0.2–0.5 (small), 0.5–0.79 (moderate), and ≥0.8 (large).

## 3. Results

The Mean and Standard Deviation (SD) for each subtest included in the KTK test battery are presented in Table 1, providing a comprehensive overview of the distribution and variability of scores within the dataset.

Table 2 presents the results of the ANCOVA analysis, which revealed significant differences between groups when controlling for age, physical activity, and BMI. Age was associated with significant differences in all variables except for walking backwards, with the largest difference observed for MQ (η*p*^2^ = 0.07, *p* < 0.01). Physical activity was linked to significant differences in walking backwards (η*p*^2^ = 0.04, *p* < 0.05), hopping for height (η*p*^2^ = 0.04, *p* < 0.01), and MQ (η*p*^2^ = 0.05, *p* < 0.05). BMI showed significant differences in walking backwards (η*p*^2^ = 0.05, *p* < 0.05) and MQ (η*p*^2^ = 0.05, *p* < 0.05).

In Table 3, the results of the ANCOVA are presented, showing differences in motor performance explained by age, gender, and BMI. Partial eta squared values indicate mostly small effects, yet several differences reached statistical significance. All three covariates were related to walking backwards (age η^2^ = 0.05, gender η^2^ = 0.02, BMI η^2^ = 0.01; all *p* < 0.01). Age also showed significant differences for jumping sideways (η^2^ = 0.11, *p* < 0.01), moving sideways (η^2^ = 0.07, *p* < 0.01), and hopping for height (η^2^ = 0.21, *p* < 0.01), confirming age as the strongest factor associated with motor performance. For the motor quotient (MQ), significant differences were observed for age (η^2^ = 0.13, *p* < 0.01), gender (η^2^ = 0.06, *p* < 0.05), and BMI (η^2^ = 0.06, *p* < 0.05), suggesting that MQ reflects the combined effect of these individual characteristics. Overall, the results indicate that, besides group differences (OPA vs. NO OPA), age, gender, and BMI account for meaningful variation in motor abilities.

## 4. Discussion

The study analyzed the difference in motor coordination in children engaged in physical activities and those who were inactive, and the gender difference between kindergarten children. Consistent with the study’s hypotheses, the key findings showed that children participating in additional physical activities achieved higher overall motor coordination scores compared to those who were inactive. The findings indicate that age, physical activity, and BMI are important determinants of motor coordination, with age showing the strongest overall effects, while physical activity and BMI contributed significantly to specific tasks and the overall motor quotient. Several studies have found that girls outperform boys in balance tasks, demonstrating statistically significant differences in motor performance. For instance, Kunz et al. [23] observed that girls consistently scored higher than boys in balance tests, with a statistically significant difference. Moreover, girls’ earlier development of fine motor control and superior proprioception has been highlighted as key factors influencing balance performance [24]. Similarly, Miller et al. [25] found significant gender differences in balance, with girls demonstrating more controlled movement patterns that likely result in better performance on tasks requiring balance and coordination. These findings collectively support the notion that girls generally outperform boys in balance-related motor skills, with statistically significant results across different studies. Therefore, our hypothesis, which stated that there would be differences in motor coordination between boys and girls, can be partially accepted, since the differences were evident primarily in balance-related tasks rather than across all motor domains. In the KTK motor coordination battery, girls often outperform boys in balance tasks, such as walking backward on a balance beam test. This difference can be attributed to several factors. Physiologically, girls tend to have a lower center of gravity and a wider pelvis, which enhances stability and balance [23]. Additionally, girls generally develop fine motor control and postural stability earlier than boys, which contributes to superior balance performance [26]. Behavioral patterns, such as girls’ tendency to engage more frequently in activities like dance and gymnastics, further enhance their balance skills [27]. Neurologically, girls also demonstrate better proprioceptive abilities and more refined body awareness, which aids in maintaining balance under dynamic conditions [24]. Finally, a more cautious approach to movement may also play a role, as girls tend to focus on maintaining stability rather than engaging in risky, high-speed movements [25]. On the other side, research results indicate that boys were better at the balance test and lateral movement, while girls were better at leg strength [28].

The difference between OPA and NO OPA in our research was statistically significant. The results showed that, in addition to group differences, significant differences in motor performance were explained by age, gender, and BMI, with age emerging as the strongest factor across most tasks. The results indicated that physically active children exhibited better motor coordination compared to their inactive peers. This suggests that regular physical activity positively influences motor coordination development in children [29]. Also, the results showed the importance of implementing such assessments to identify motor coordination deficits and promote physical activity among children [30]. A study comparing motor coordination in children engaged in multisport activities, swimming, and inactive children found significant differences in all KTK subtests, including walking backward, hopping for height, jumping sideways, and moving sideways. The results showed that physically active children had higher motor coordination scores compared to inactive children [4]. Another study confirmed this result by researching differences in motor skills between active and inactive children in elementary school. While this study primarily used BOT-2 and TGMD-2 tests, it highlighted the importance of physical activity in enhancing motor skills [31]. Therefore, the hypothesis that children engaged in organized sports activities would achieve higher scores on the KTK motor coordination test compared to those not involved in such activities is accepted. Additional physical activity programs can significantly improve the abilities of preschool children. For instance, boys showed a noticeable improvement in their flexibility test results, whereas girls did not. This may be due to the fact that girls generally have a naturally higher level of flexibility from the start [32].

In our study, we utilized the KTK exclusively for children in kindergarten to assess their gross motor coordination at an early developmental stage. However, this study has several key limitations. First, physical activity was not measured objectively, and we used only one test to assess motor coordination, which may affect the accuracy and validity of the results, as subjective measurements can introduce errors in assessing activity levels. Also, the conclusions relate to only one part of the country, which limits the possibility of generalization to the entire population, so additional analysis is needed that includes data from other parts of the country and will thus provide more precise results on children’s motor coordination. Finally, the participants came from different sports and sports schools, with different experiences, i.e., the period when they joined an activity, which creates heterogeneity in the group and makes it difficult to draw conclusions about which activity is best, as the results may be specific to individual sports. Given these limitations, further research is needed that will include objective measures of physical activity, use additional tests to assess children’s motor coordination, include a wider population, and group different sports activities in order to determine which activity most contributes to the development of coordination in preschool children.

## 5. Conclusions

The results indicated that physically active children exhibited better motor coordination compared to their inactive peers. This suggests that regular physical activity positively influences motor coordination development in children. Also, results showed that girls were better at walking backwards and MQ than boys in overall motor coordination. Factors such as greater engagement in activities that emphasize coordination, as well as potential differences in neuromuscular development, may contribute to these results. However, further analysis is needed to explore whether these differences persist as children grow older and whether they are influenced by varying levels of physical activity between genders and include a wider population. Also, group different sports activities in order to determine which activity most contributes to the development of coordination in preschool children. Expanding the research to include a more diverse, rural location and a larger population sample would improve the generalizability of the findings. Moreover, future research should be longitudinal studies that track developmental changes over time and the influence of different physical activities on the development of motor abilities.

## Figures and Tables

**Table 1:** Descriptive statistics.

Variable	Boys		Girls	
	Mean	SD	Mean	SD
Age	5.58	0.49	5.57	0.25
Height	119.82	7.95	118.94	8.19
Weight	23.66	5.45	23.84	9.29
BMI	17.72	19.39	16.6	6.06
Walking backwards	32.17	10.64	35.27	10.67
Jumping sideways	41.73	14.66	42.51	14.05
Moving sideways	12.97	3.37	12.65	4.42
Hopping for height	25.549	7.87	24.64	6.54

**Table 2:** Results of motor coordination tests in preschoolers according to gender.

Variable	*Boys*	*Girls*	ANCOVA		
	Mean ± SD	Mean ± SD	Age	Physical Activity	BMI
			η^2^	η^2^	η^2^
Walking backwards	32.17 ± 10.64	35.27 ± 10.67	0.05 **	0.04 *	0.05 *
Jumping sideways	41.73 ± 14.66	42.51 ± 14.05	0.03 **	0.01	0.01
Moving sideways	12.97 ± 3.37	12.65 ± 4.43	0.04 **	0.01	0.02
Hopping for height	25.54 ± 7.88	24.64 ± 6.54	0.04 **	0.04 **	0.01
MQ	100.42 ± 21.74	105.34 ± 20.78	0.07 **	0.05 *	0.05 *

Legend: *—statistical significant (*p* ≤ 0.05); **—statistical significant (*p* ≤ 0.01).

**Table 3:** Results of motor coordination tests in preschoolers according to physical activity.

Variable	OPA	NO OPA	ANCOVA		
	Mean ± SD	Mean ± SD	Age	Gender	BMI
			η^2^	η^2^	η^2^
Walking backwards	35 ± 10.11	32.77 ± 11.13	0.05 **	0.02 **	0.01 **
Jumping sideways	45.41 ± 14.05	39.7 ± 14.11	0.11 **	0.02	0.01
Moving sideways	13.55 ± 4.71	12.27 ± 13.16	0.07 **	0.01	0.01
Hopping for height	26.58 ± 7.27	24.01 ± 7.06	0.21 **	0.02	0.01
MQ	107.68 ± 20.21	99.35 ± 21.58	0.13 **	0.06 *	0.06 *

Legend: *—statistical significant (*p* ≤ 0.05); **—statistical significant (*p* ≤ 0.01).

## Data Availability

The data presented in this study are available on request from the corresponding author. Due to the involvement of minors in this study, the dataset cannot be made publicly available to protect participants’ privacy and confidentiality. Data can be obtained upon reasonable request by contacting the corresponding author.
